# Preparation and properties of a photocrosslinked MCl_*n*_-doped PDMA-*g*-PSMA hydrogel

**DOI:** 10.1039/d2ra07079k

**Published:** 2023-01-18

**Authors:** Zhaocong Chen, Hongyan Wu, Jialei Fei, Qinghua Li, Ruian Ni, Yanzhao Qiu, Danning Yang, Lu Yu

**Affiliations:** a School of Environmental Science and Engineering, Nanjing University of Information Science and Technology Nanjing 210044 PR China; b Institute of Advanced Materials and Flexible Electronics (IAMFE), School of Chemistry and Materials Science, Nanjing University of Information Science and Technology Nanjing 210044 PR China wuhy2009@nuist.edu.cn

## Abstract

To treat damaged joint areas, photocrosslinked hydrophobically associating PDMA-*g*-PSMA hydrogels can act as mild and easily regulated materials due to their rich pore structure, which have been widely applied in articular cartilage replacement research. In this study, the effects of ADS–MCl_*n*_ (ADS–NaCl, ADS–MgCl_2_ and ADS–CaCl_2_) doping systems on the micro morphology, mechanical, self-healing, and friction properties and cytotoxicity of PDMA-*g*-PSMA hydrogels were studied. The results showed that the solubilization behavior of the ADS–MCl_*n*_ ionic micelles affected the hydrophobic association stability, thereby changing the toughness, self-healing and friction properties of the hydrogel. Ca^2+^-doping resulted in the crystallization and precipitation of the anionic surfactants, destroying the solubilization ability of the ionic micelles for the hydrophobic units, and thus hydrogels with high hardness, low toughness and no self-healing function were obtained. Doping with Na^+^ greatly improved the dissolving power of the ADS micelles for SMA, yielding PDMA-*g*-PSMA hydrogels with good mechanical strength and good self-healing ability. However, in this case, a drawback is that the Na^+^-doped system will lose its components during the swelling process, leading to the degradation of its self-healing performance. Interestingly, Mg^2+^ doping resulted in the formation of highly stable ADS micellar aggregates, and then PDMA-*g*-PSMA hydrogels with a lower friction coefficient (0.023), less wear (35.0 mg), higher elongation at break and 100% self-healing efficiency were obtained. The hydrogel products obtained from the three doping systems all exhibited good biocompatibility. Our research provides important guidelines for the design and preparation of anti-friction artificial articular cartilage.

## Abstract 2

Na^+^ or Mg^2+^ doping can improve the elongation at break, equilibrium swelling ratio and self-healing efficiency of PDMA-*g*-PSMA hydrogels. Among them, MgCl_2_-doped PDMA-*g*-PSMA has better shape stability, lower friction coefficient and lower wear loss.

## Graphical abstract

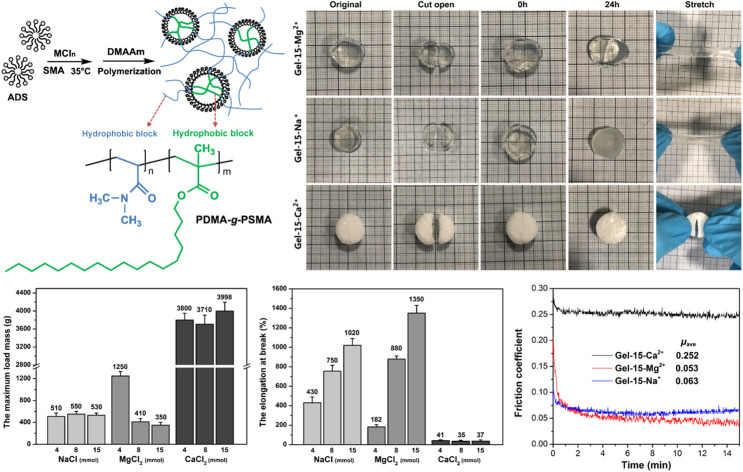

## Introduction

Articular cartilage is a dense, translucent, non-vascular, non-neural white connective tissue covering the articular surface,^1,2^ providing a low-friction and wear-resistant sliding surface, which reduces the stress and vibrations borne by the bone.^3,4^ Articular cartilage is characterized by multiple layers and anisotropy.^5,6^ Superficial cartilage has low tensile strength and stiffness and a compressive Young's modulus of 280 ± 160 kPa, whereas deep cartilage has high compressive strength and a compressive Young's modulus of 730 ± 260 kPa.^7,8^ The friction coefficient of these two types of cartilage is extremely low (0.001–0.03).^8^ In addition, articular cartilage has the characteristic of withstanding mechanical stress without undergoing permanent deformation. Also, due to the poor proliferation ability of chondrocytes, cartilage tissue has no blood vessels and low catabolic ability to pathological intermediates, and once the articular cartilage is damaged, it is difficult to repair itself.^9^ Therefore, it is necessary to treat cartilage injury by surgery or tissue engineering in clinic.^10^ In this case, the development of tissue substitutes *in vitro* has been proven to be a feasible strategy to assist the regeneration and reconstruction of damaged cartilage.^11–13^

Hydrogels are three-dimensional network materials formed by hydrophilic polymers or macromolecules with unique physical and chemical properties. Similar to the natural extracellular matrix, hydrogels have a porous structure, which can achieve maximum swelling in the aqueous phase and act as a scaffold for the proliferation and differentiation of chondrocytes.^14^ Simultaneously, they can be molded in many ways, *e.g.*, injecting or placing into the injured joint area to form the required three-dimensional network structure *in situ*.^15^ Therefore, hydrogels are preferred materials for the preparation of artificial articular cartilage. A variety of hydrogels such as poly(ethylene oxide terephthalate)/poly(butylene terephthalate) (PEOT/PBT),^16^ poly(ε-caprolactone) (PCL),^17,18^ collagen,^19^ chondroitin sulfate (CS), and gelatin^20^ has been reported as alternatives to articular cartilage. Among the various hydrogel-forming methods, photocrosslinking is a popular scheme for the preparation of artificial cartilage and bone repair scaffolds because of its mild polymerization conditions, low toxicity, non-contact and easy regulation. It has been reported that photocrosslinked hydrogels are suitable for the encapsulation of chondrocytes (CH) and bone marrow stem cells (BMSCs),^21,22^ and also the *in situ* preparation of cartilage substitute tissues.^23^ Additionally, the rapid photocrosslinking process does not have the obvious toxic and side effects on bone cells and organisms.

During the preparation of artificial articular cartilage, in addition to considering their biocompatibility, degradability, cell adhesion and effects on cell differentiation and cartilage formation,^24^ it is also very important to simulate the mechanical property and lubrication properties of natural cartilage. Trucco *et al.*^23^ prepared a photocrosslinked bilayer composed of gellan gum (GG) and poly(ethylene glycol)diacrylate (PEGDA) to simulate the mechanical and lubrication properties of the surface and deep layers of articular cartilage. The results showed that the surface friction of the GG/PEGDA10 hydrogels could be reduced by introducing GO, which was reduced to 0.031 under 10 N load and PBS as the lubricant. Freeman *et al.*^25^ studied the effects of lubricant, load weight, degree of crosslinking, and degree of hydration on the tribological behavior of poly(2-hydroxyethyl)methacrylate (PHEMA) hydrogels. The results showed that the friction coefficients of the PHEMA hydrogels were in the range of 0.02 to 1.7. Increasing the applied load, decreasing the crosslink density of the hydrogel, or increasing the degree of hydration of the hydrogel resulted in an increase in wear. Shin *et al.*^26^ prepared photocrosslinked gellan gum methacrylate/gelatin methacrylamide (GGMA/GelMA) double network hydrogel-engineered cartilage. The compressive strength of the material was 6.9 MPa, which is similar to that of natural cartilage. Levett *et al.*^27^ modified gelatin-methacrylamide (Gel-MA) hydrogels with a small amount of hyaluronic acid methacrylate (HA-MA) and chondroitin sulfate methacrylate (CS-MA) *via* photocrosslinking reaction. The formation rate of the modified cartilage increased and its mechanical properties were improved.

Introducing an effective energy dissipation mechanism in a hydrogel can improve the resistance of the material to crack propagation^28,29^ and enhance the mechanical strength of the artificial cartilage. Selecting hydrophobic associations with the finite lifetime and reversible fracture-crosslinking process to construct polymer networks has been demonstrated to improve the overall viscoelastic dissipation of materials,^30^ resulting in artificial cartilage with high toughness and high self-healing efficiency. In our previous work, we have reported the self-healing and mechanical properties of PDMA-based^31^ and PNIPAM-based^32^ hydrophobically associating hydrogels. The hydrophobic monomer SMA was first solubilized in the aqueous phase by ADS–NaCl micellar solution, and then grafted in the hydrophilic polymer network by free radical polymerization. Due to the high local concentration of hydrophobic monomers in the micelles, the hydrophobic segments are randomly distributed along the hydrophilic polymer backbone and have the characteristics of reversible breaking-crosslinking. The physically cross-linked hydrogels obtained by this protocol exhibited remarkable non-ergodicity, self-healing, and high toughness, and were insoluble in water but soluble in ADS solution. Studies have shown that the type of hydrophobic segment and the concentration of surfactant have a significant effect on the lifetime and strength of hydrophobic association.^30,33,34^ Understanding the regulation mechanisms of the hydrophobic association has important guiding significance in the design and preparation of high strength and high self-healing hydrogel artificial cartilage.

Herein, based on our previous work,^31,32^ UV-crosslinked hydrophobically associating PDMA-*g*-PSMA hydrogels were prepared using NaCl/MgCl_2_/CaCl_2_ as doped inorganic salts, ADS as ionic micelles and Irgacure-2959 as the photoinitiator. The morphology, swelling behavior, mechanical property, self-healing efficiency, hydrophilicity and hydrophobicity, friction, wear resistance and cytotoxicity of the hydrogels were investigated to explore how the physical and chemical properties of the PDMA-*g*-PSMA hydrogels were controlled by the doping components of inorganic salts by affecting the solubilization behavior of ionic micelles. These results can provide a novel idea for the design of artificial hydrogel articular cartilage.

## Materials and methods

### Materials

Ammonium lauryl sulfate (ADS, 70%) was purchased from Yousuo Chemical Reagent Co. (Qingdao, China). Stearyl methacrylate (SMA, 98%) and *N*,*N*-dimethylacrylamide (DMAAm, 99.9%) were purchased from Sigma-Aldrich (Saint Louis, MO, USA). *N*,*N*,*N*′,*N*′-Tetramethylethylenediamine (TEMED), sodium chloride, magnesium chloride, calcium chloride, and ethanol were of analytical grade and purchased from Nanjing Chemical Reagent Co. (Nanjing, China). 2-Hydroxy-4′-(2-hydroxyethoxy)-2-methylpropiophenone (trade name: Irgacure-2959) was provided by BASF Co., Ltd. (Germany). All reagents were used as received.

### Synthesis of MCl_*n*_-doped PDMA-*g*-PSMA

**Fig. 1:**
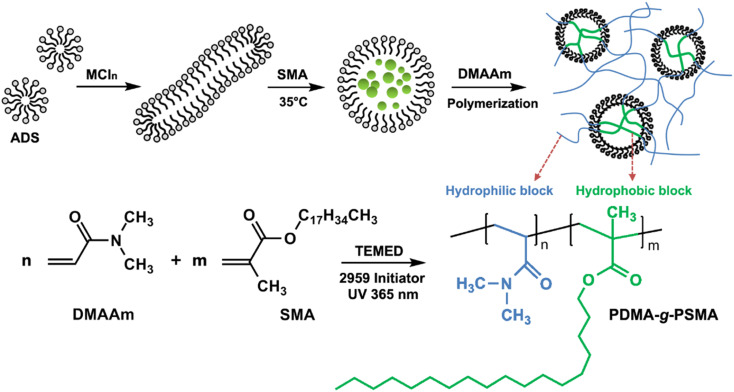
Schematic diagram of the preparation of MCl_*n*_-doped PDMA-*g*-PSMA hydrogel.

Briefly, 7.4 mmol of ADS and 15.0 mmol of inorganic salt MCl_*n*_ (sodium chloride, magnesium chloride, or calcium chloride) were dissolved in the distilled water at room temperature. Subsequently, 0.84 mmol SMA was added and stirred in a water bath at 35 °C for 2 h, and then 42.7 mmol DMAAm was added and stirred. Then, 0.5 mmol of TEMED and 0.45 mmol of Irgacure-2959 UV initiator were added and stirred well. The reaction solution was transferred to a glass mold and nitrogen was introduced to remove oxygen from the solution. Finally, the solution was irradiated by a 365 nm UV light source to obtain the MCl_*n*_-doped PDMA-*g*-PSMA hydrogel. A schematic diagram of the process for the preparation of the PDMA-*g*-PSMA hydrogel is shown in Fig. 1.

The PDMA-*g*-PSMA hydrogels doped with 15 mmol NaCl, 15 mmol MgCl_2_ and 15 mmol CaCl_2_ were named Gel–15-Na^+^, Gel–15-Mg^2+^ and Gel–15-Ca^2+^, respectively. Besides, PDMA-*g*-PSMA hydrogels with 8 mmol and 4 mmol inorganic salts were prepared to investigate the effect of the inorganic salt on the physical and chemical properties of the hydrogels. Likewise, they were designated as Gel–8-Na^+^, Gel–8-Mg^2+^, Gel–8-Ca^2+^, Gel–4-Na^+^, Gel–4-Mg^2+^ and Gel–4-Ca^2+^.

### Quantification of the solubilization of SMA in ADS–MCl_*n*_ solutions

The test was performed using a Cary-50 UV-vis spectrophotometer (Varian, USA). Briefly, 2.10 g (7.4 mmol) of ADS and inorganic salt (NaCl, MgCl_2_, or CaCl_2_) were dissolved in 30.0 mL of distilled water, and then SMA added and stirred in a water bath at 35 °C for 24 h. The transmittance of the SMA–ADS–MCl_*n*_ solution at 500 nm was tested, and the relationship between transmittance and SMA quality was plotted. The solubility of SMA at different inorganic salt concentrations was determined based on the inflection point of the curve.^33^

### Dynamic light scattering (DLS)

The hydrodynamic correlation length (*ξ*_H_) of the ADS micelle solution was measured using a BI-200SM laser light scattering spectrometer (Brookhaven, USA) equipped with a digital detector (BI-PAD) and a semiconductor laser light source operating at 532 nm. The test temperature was 35 °C.

### Fourier infrared spectroscopy (FT-IR)

FTIR was carried out using a Nicolet iS-10 Fourier infrared spectrometer (Thermo Fisher Scientific, USA) in the scanning range of 500 cm^−1^ to 4000 cm^−1^ with the resolution of 4 cm^−1^. The MCl_*n*_-doped PDMA-*g*-PSMA hydrogels were frozen in liquid nitrogen for 4 h before testing. Then, they were dried for 24 h using an FD-1C-50 freeze dryer (Boyikang, China). The dried samples were sliced, and then tested.

### Scanning electron microscopy (SEM)

A JSM-7600F field-emission scanning electron microscope (JEOL, Japan) was used to characterize the morphology and structure of the MCl_*n*_-doped PDMA-*g*-PSMA hydrogels. Before the test, the hydrogels were soaked in deionized water for 3 days to reach swelling equilibrium. Then, they were frozen in liquid nitrogen for 4 h and dried using a freeze dryer for 24 h. The dried samples were sliced and sprayed with gold, and finally observed by SEM.

### Equilibrium swelling ratio determination (ESR)

The ESR of the MCl_*n*_-doped PDMA-*g*-PSMA hydrogels at room temperature was determined *via* the gravimetric method. Firstly, the freshly prepared hydrogels were cut into disks with a diameter of 13 mm and thickness of 5 mm and vacuum dried to a constant weight at 60 °C. Then, the hydrogels were weighed to obtain their dry weight (*W*_dry_). The dried hydrogels were immersed in deionized water at 20 °C and swelled to constant weight. Subsequently, they were taken out, quickly dried with filter paper and weighed to obtain their weight after swelling equilibrium (*W*_e_) was reached. The equilibrium swelling ratio (ESR) of the hydrogels can be calculated according to the following formula:ESR = (*W*_e_ − *W*_dry_)/*W*_dry_

### Thermal gravimetric analysis (TGA)

TGA was performed on a Pyris-1 TGA instrument (PerkinElmer, USA). Before the test, the MCl_*n*_-doped PDMA-*g*-PSMA hydrogels were soaked in deionized water for 3 days to reach swelling equilibrium. Then, they were taken out, quickly dried with filter paper, and heated at 10 °C min^−1^ from 20 °C to 600 °C under a nitrogen atmosphere.

### Mechanical properties

**Fig. 2:**
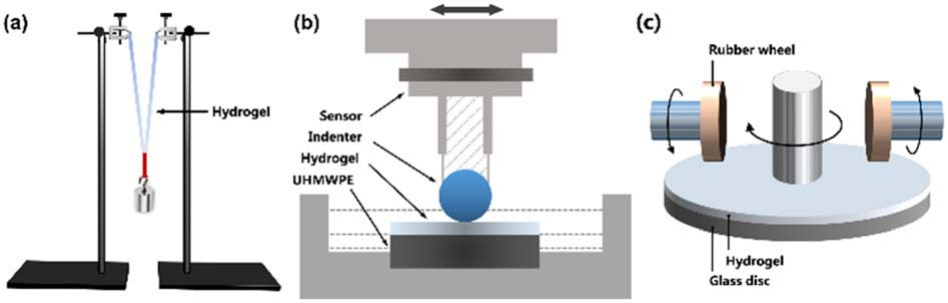
(a) Schematic diagram of load test of hydrogel. (b) Schematic diagram of hydrogel surface friction coefficient test device. (c) Schematic diagram of hydrogel abrasion resistance test device.

The mechanical strength of the MCl_*n*_-doped PDMA-*g*-PSMA hydrogels was measured by a hanging load experiment at 70% humidity (Fig. 2a). Firstly, the hydrogel samples were cut into cylinders with a length of 50 mm and diameter of 13 mm. Then, the hydrogel cylinders were folded and weights hung on them. The weight of the load was slowly increased until the hydrogel broke. At this time, the loaded weight was the maximum load mass (*M*_max_) of the hydrogel. The length change of the hydrogel after loading the weight with a camera was recorded and the breaking elongation, *e*, of the hydrogel was calculated according to the following formula:*e* = (*L*_a_ − *L*_o_)/*L*_o_where *e* is the elongation at break, *L*_o_ is the original length of the sample, and *L*_a_ is the length when the sample is broken. The measurement was repeated five times for each hydrogel sample.

### Tensile-recovery test

The hydrogel sample was cut into a cylinder with a length of 30 mm and diameter of 13 mm. The hydrogel was slowly stretched to 150 mm at 70% humidity and 25 °C for 2 min. Then, the stretching was released and the hydrogel recovered at room temperature for 2 min. The stretching-recovery cycle was repeated 10 times for each sample and the hydrogel length after recovery was measured. All samples were tested 3 times in parallel.

### Self-healing performance

The hydrogel samples were cut into discs with a thickness of 5 mm and diameter of 13 mm. Then, a weight was hung in the middle of the hydrogel and the mass of the weight slowly increased until the hydrogel broke. The mass loaded was counted as the initial maximum load, *M*_o_, of the hydrogel. Taking the hydrogel of the same size, a disk was in the middle of it with a knife. Subsequently, the cut surface was reattached and the hydrogel was placed in an environment with 70% humidity and 25 °C for 24 h. After curing, a weight was hung at the fractured position of the hydrogel and the mass of the weight slowly increased until the hydrogel broke again. The loaded mass was counted as the maximum load of the hydrogel after self-healing (*M*_sh_). The self-healing efficiency (%) of the hydrogel is defined as *M*_sh_/*M*_max_. All samples were tested 5 times in parallel.

### Uniaxial compression test

A Zwick Roell testing machine equipped with a 10 N load cell was used for the cyclic compression test of the hydrogels. Firstly, the hydrogel was cut into a cylinder with a diameter of about 4 mm and length of about 5 mm, and then placed between the plates of the instrument. Before the test, an initial compression contact of 0.004 ± 0.003 N was applied to ensure full contact between the gel and the plate. The cyclic test was carried out, where the compression step was the conducted at a constant crosshead speed of 5 mm min^−1^, and retracted to zero displacement immediately after reaching the maximum load. Then, it was held for 2 min, and the next compression cycle was conducted. The compression stress, *σ*_Nom_, and strain, *λ* (*λ* = deformation length/initial length) were recorded. The test ambient temperature was 25 °C.

### Contact angle

The water contact angle of the MCl_*n*_-doped PDMA-*g*-PSMA hydrogel was characterized using a JC2000D1 contact angle measuring instrument (Powereach, China). Before the test, all samples were soaked in deionized water for 3 days to achieve swelling equilibrium and dried with filter paper.

### Tribological properties

The tribological properties of the MCl_*n*_-doped PDMA-*g*-PSMA hydrogels were tested using a CFT-1 material surface performance tester (Zhongkekaihua, China, Fig. 2b). The diameter of the silicon nitride grinding ball was 4 mm, the friction stroke as 5 mm, the sliding speed was 200 rpm, the load was 3 N, and the friction test time was 15 min. To simulate the lubricating function of body fluid on articular cartilage, the surface friction coefficients of each sample without lubricant and with distilled water as the lubricant were tested.

### Abrasion resistance test

The abrasion resistance of MCl_*n*_-doped PDMA-*g*-PSMA was characterized using the rotating abrasive rubber wheel method. Firstly, the mixed monomer solution was uniformly coated on the surface of a glass disk with a diameter of 10 cm, and then irradiated with 365 nm UV light for 8 min to obtain the PDMA-*g*-PSMA hydrogel film (thickness of 52 ± 5 μm). The glass disk coated with the hydrogel was installed on a BGD523 film abrasion tester (Biugde, China). Under the conditions of 500 g load, 20 °C and 85% humidity, the abrasion experiment was carried out with a CS-10 abrasive rubber wheel (Fig. 2c). After running for 100, 200, 300, 400 and 500 revolutions, the mass loss of hydrogel was recorded.

### Cytotoxicity test

The standard CCK-8 (Cell Counting Kit-8, Kumamoto, Japan) assay was used to study the cytotoxicity of the MCl_*n*_-doped PDMA-*g*-PSMA hydrogels. Firstly, the hydrogel was subjected to ultraviolet sterilization and soaked in distilled water to swell for 5 days, and the extract collected. Mouse L929 fibroblasts were cultured for 24 h with RPMI containing 10 vol% fetal bovine serum, 4.5 g L^−1^ glucose, and 100 units per mL penicillin (100 μL medium, 10^4^ cells). The hydrogel extract was added to the culture dish (the same amount of PBS was added to the control group), and the culture was continued for 24 h (27 °C). Then, the media and samples were removed, 100 μL medium and 10 μL CCK-8 solution added to each Petri dish, and culture continued for 2 h. The above-mentioned procedure was repeated without the addition of L929 fibroblasts to obtain a blank group. A UV-vis spectrophotometer was used to measure the absorbance of different culture groups at 450 nm, and the following formula used to calculate the cell survival rate:Cell survival rate (%) = (*A*_*t*_ − *A*_0_)/(*A*_PBS_ − *A*_0_) × 100%where *A*_*t*_ is the absorbance of the hydrogel extract culture group, *A*_PBS_ is the absorbance of the PBS culture group and *A*_0_ is the absorbance of the blank group.

Finally, the L929 cells in each culture group were stained with 10 μL diluted calcein/propidium iodide solution for 30 min and observed and recorded by an LSM 800 confocal laser microscope.

## Results and discussion

### Solubilization of hydrophobic monomers by ionic micelles

**Fig. 3:**
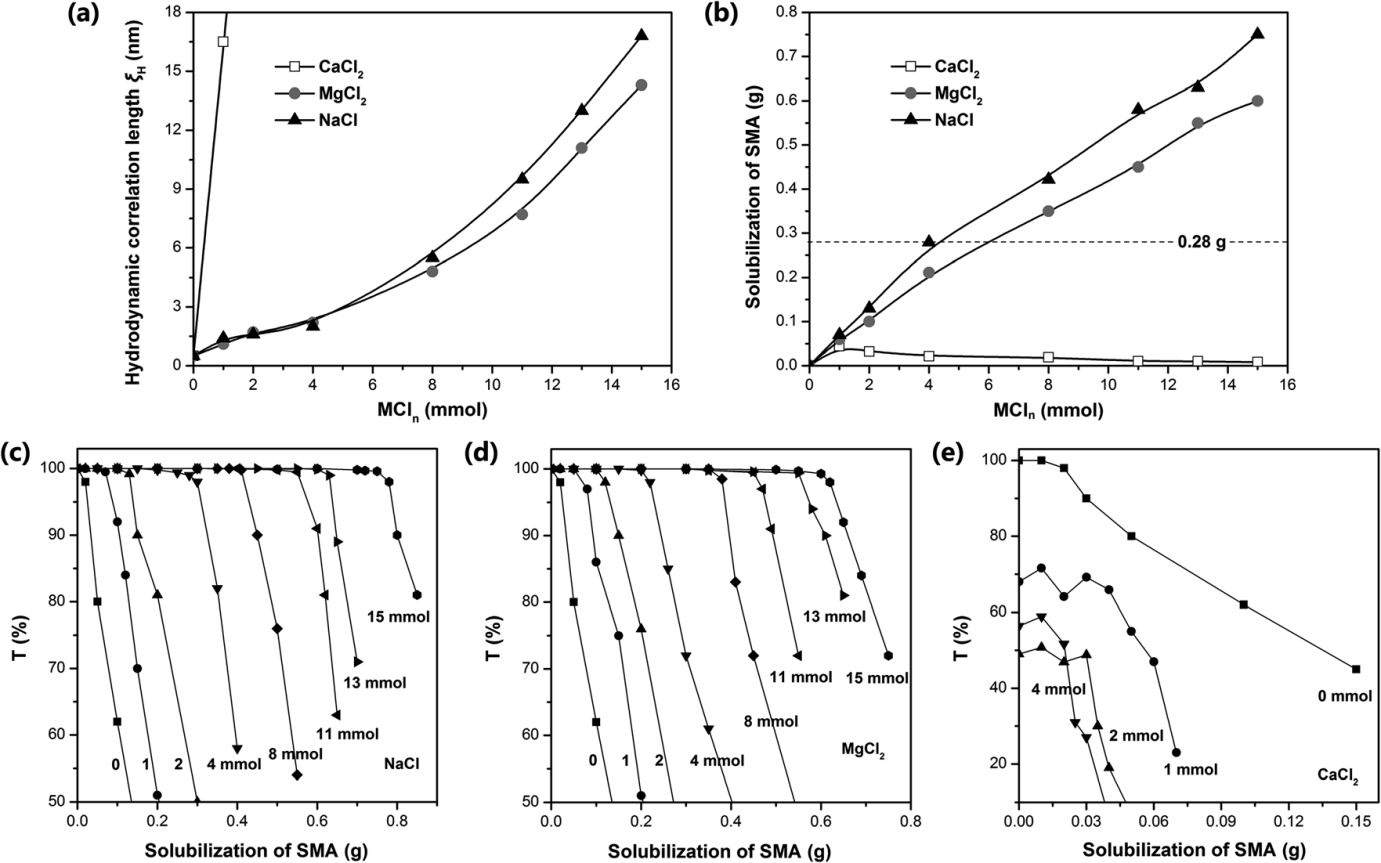
(a) Hydrodynamic correlation length (*ξ*_H_) of ADS micellar solution at different MCl_*n*_ dosages (30 mL distilled water, 7.4 mmol ADS, 35 °C). (b) Amount of SMA dissolved in ADS–MCl_*n*_ solution under different MCl_*n*_ concentrations (30 mL distilled water, 7.4 mmol ADS, 35 °C). Transmittance curve of (c) SMA–ADS–NaCl, (d) SMA–ADS–MgCl_2_, and (e) SMA–ADS–CaCl_2_ solution at 500 nm with the addition of different MCl_*n*_ (35 °C).

Dynamic light scattering (DLS) was used to measure the hydrodynamic correlation length (*ξ*_H_) of the ADS micelle solutions with different MCl_*n*_ contents. The test results show (Fig. 3a) that at the initial stage of adding Na^+^ and Mg^2+^, *ξ*_H_ increased slowly. When the amount of NaCl and MgCl_2_ exceeded 0.4 mmol, the salt concentration continued to increase, causing *ξ*_H_ to increase rapidly and the micelle size to increase significantly. It has been reported that the addition of counterions can significantly reduce the critical micelle concentration (CMC) of ionic surfactants and increase the aggregation number of micelles.^35–39^ In this case, Na^+^ and Mg^2+^ will combine with the micelles, making the diffusion double layer structure of ionic micelles thinner. Simultaneously, they will weaken the electrical repulsion between the ion head groups of the surfactant, promoting the micelles to grow from spherical to rod-shaped, large cylindrical aggregates or flexible worm-shaped micelles.^30^ After the addition of SMA monomer, the micellar aggregate will be further transformed into an oil-in-water microemulsion with SMA as the core to achieve the solubilization of SMA (as shown in Fig. 1). Compared with Na^+^, Mg^2+^ has more charges, smaller hydration radius, stronger binding force with ADS micelles, and more significant reduction effect on CMC.^37^ Simultaneously, Mg^2+^ can combine with two ADS head groups to reduce the area occupied by a single head group, which is more conducive to the transition of micelles from spherical to rod-like.^40,41^

Although the effects of Na^+^ and Mg^2+^ on CMC and micelle growth are relatively clear, the solubilization efficiency of SMA could only be determined by experiments due to the complexity of the aggregation structure of the ADS–MCl_*n*_ micelles. Accordingly, the transmittance (500 nm) of the SMA–ADS–MCl_*n*_ micelle solution was characterized using a UV-vis spectrophotometer. The relationship curve between the transmissivity *T* and the SMA quality is shown in Fig. 3c–e, where the solubility of SMA in the ADS–MCl_*n*_ micelles was determined through the inflexion point of the curve. The results show that the solubility of SMA was the highest in the ADS–NaCl ionic micelle solution (see Fig. 3b), followed by ADS–MgCl_2_. The solubility of SMA increased with an increase in the content of NaCl and MgCl_2_. When the amount of NaCl added was up to 4 mmol, 8 mmol and 15 mmol, the dissolved amount of SMA was 0.28 g, 0.42 g and 0.75 g, respectively. When the amount of MgCl_2_ was 4 mmol, 8 mmol and 15 mmol, the dissolved amount of SMA was 0.21 g, 0.35 g and 0.60 g, respectively. Thus, the counterions (Na^+^ and Mg^2+^) introduced by external electrolyte were the main factors affecting the solubilization behavior of the ADS solution.

The solubilization of the ADS–MgCl_2_ system for SMA monomer was lower than that of the ADS–NaCl system at the concentration of ADS selected herein. This may be due to the higher stability and rigidity of the ADS–MgCl_2_ ionic micelles, which restricted the transformation of the micellar aggregates into SMA–ADS–MgCl_2_ microemulsions. The ADS–CaCl_2_ system exhibited the weakest solubility for SMA (less than 0.045 g). This is because CaCl_2_ and ADS can form the water-insoluble calcium dodecyl sulfate. As the content of CaCl_2_ increases, the number of ADS micelles will decrease, leading to a decrease in SMA solubility.

**Fig. 4:**
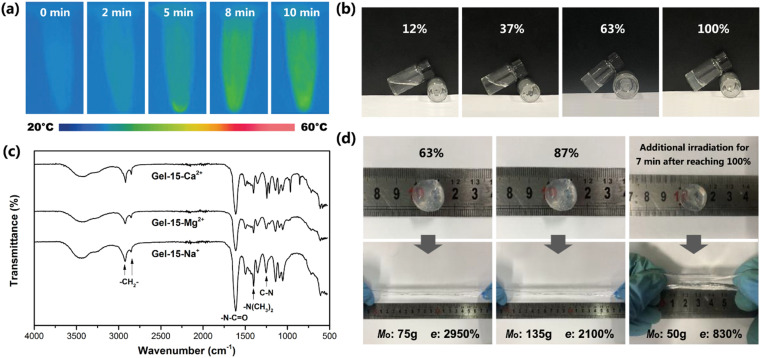
(a) Infrared thermal image of Gel–15-Mg^2+^ mixed monomer solution under different laser irradiation times. (b) Appearance of Gel–15-Mg^2+^ hydrogel under different reaction degrees. (c) FTIR spectra of Gel–15-Na^+^, Gel–15-Mg^2+^ and Gel–15-Ca^2+^ hydrogels after photocrosslinking reaction for 8 min. (d) Deformation of Gel–15-Mg^2+^ hydrogel disc after loading and its maximum mass, *M*_o_, and elongation at break, *e*, under different reaction degrees.

By comparing the addition amount of SMA (0.28 g, dotted line in Fig. 3b) with its solubility, it can be found that Gel–4-Na^+^, Gel–8-Na^+^, Gel–15-Na^+^, Gel–8-Mg^2+^ and Gel–15-Mg^2+^ were prepared under the condition that SMA was completely dissolved, and the obtained hydrogels were colorless and transparent (Fig. 4b). In contrast, Gel–4-Mg^2+^, Gel–4-Ca^2+^, Gel–8-Ca^2+^ and Gel–15-Ca^2+^ were polymerized without the complete solubilization of SMA by the micelles. At this time, the undissolved SMA was suspended in the aqueous solution in the form of large particle size droplets to participate in polymerization. Thus, the obtained hydrogels were milky white with relatively poor transparency.

### Photocrosslinking process and product structure

The exothermic process of photocrosslinking was tracked using an infrared thermal imager. Fig. 4a shows the infrared thermal images of the Gel–15-Mg^2+^ mixed monomer solution under different laser irradiation times. Before illumination, the temperature of the solution was 26.4 °C. After laser irradiation, the radical polymerization changed to exothermic, and the solution temperature gradually increased to 38.2 °C. By observing the heating process, it was found that the heating rate decreased rapidly after 8 min, which indicates that the monomer polymerization tended to be complete after 8 min of illumination.

The photocrosslinking process of PDMA-*g*-PSMA was characterized by tracking the characteristic absorption of the tertiary amide groups *via* FT-IR. Before photocrosslinking, the characteristic absorption of the tertiary amide group of the DMAAm monomer appeared at 1648 cm^−1^. After ultraviolet light (365 nm) irradiation, due to the disappearance of the C=C conjugation effect and the formation of hydrogen bonds, the characteristic absorption of the tertiary amide group in the PDMA chain segment shifted to 1612 cm^−1^.^42,43^ The reaction degree of the monomer can be roughly estimated based on the ratio of the absorption peak intensity of 1648 cm^−1^ to 1612 cm^−1^. Taking the Gel–15-Mg^2+^ hydrogel system as an example, when the monomer reaction reached about 37%, the system began to gel and the fluidity decreased (Fig. 4b). When the irradiation time reached 8 min, the photocrosslinking of the Gel–15-Mg^2+^ system tended to be complete. At this time, the characteristic absorption of the DMAAm monomer at 1648 cm^−1^ disappeared completely, and the C=C characteristic absorption of the DMAAm and SMA monomers at 1602 cm^−1^ and 1630 cm^−1^, respectively, also disappeared completely. Fig. 4c shows that both the homogeneous Gel–15-Na^+^ and Gel–15-Mg^2+^ systems and heterogeneous Gel–15-Ca^2+^ systems yielded polymerization products through photocrosslinking reaction, and their infrared spectra were similar. The characteristic absorption bands at 2922 cm^−1^ and 2852 cm^−1^ are attributed to the asymmetric and symmetric stretching vibrations of CH_2_ in the PSMA, respectively.^44^ The characteristic absorption bands at 1612 cm^−1^ and 1401 cm^−1^ correspond to the C=O stretching vibration (amide I band) of the tertiary amide group in the PDMA chain segment and the deformation vibration of (CH_3_)_2_–N–, respectively.^45,46^ The characteristic absorption at 1253 cm^−1^ is attributed to the C–N stretching vibration.^42,47^

In addition, the experiment showed that the mechanical properties of the PDMA-*g*-PSMA hydrogel were significantly different at the different polymerization stages. Taking the Gel–15-Mg^2+^ hydrogel system as an example, in the initial stage of polymerization, the hydrogel had a high elongation at break and low mechanical strength. When the reaction time was prolonged, the elastic modulus of the PDMA-*g*-PSMA hydrogel gradually increased, its elongation at break (*e*) gradually decreased, and its heavy load mass (*M*_o_) rapidly increased initially, and then slightly declined (Fig. 4d). When the reaction degree was 87%, the mass *M*_o_ that could be loaded on the Gel–15-Mg^2+^ hydrogel disc reached the peak value (135 g). When the irradiation time reached 8 min, the photocrosslinking of the Gel–15-Mg^2+^ system tended to be complete, and *M*_o_ slightly decreased to 125 g. Prolonged laser irradiation can cause the aging of hydrogels.^48^ The experimental results showed that when the photocrosslinking reaction was completed, the *M*_o_ of the hydrogel decreased rapidly to 50 g and the elongation at break decreased to 830% after additional illumination for 7 min (Fig. 4d). Therefore, the illumination time must be controlled reasonably during the preparation of photocrosslinked PDMA-*g*-PSMA hydrogels.

**Fig. 5:**
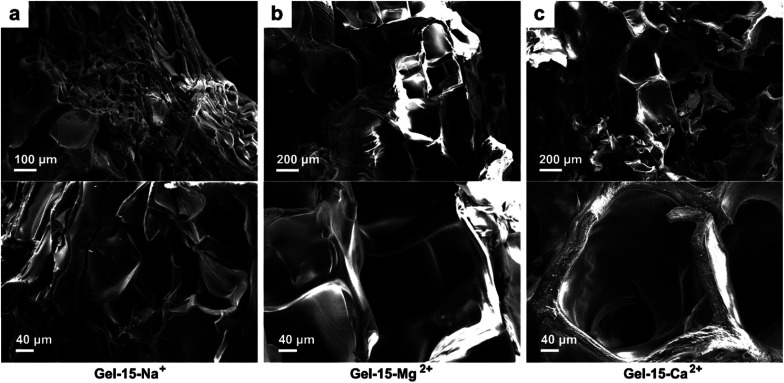
SEM photos of (a) Gel–15-Na^+^, (b) Gel–15-Mg^2+^, and (c) Gel–15-Ca^2+^ hydrogels after lyophilization.

The micro morphology of the MCl_*n*_-doped PDMA-*g*-PSMA hydrogel was observed by SEM. Fig. 5 shows the SEM photographs of the Gel–15-Na^+^, Gel–15-Mg^2+^, and Gel–15-Ca^2+^ hydrogels after lyophilization. It can be found that the PDMA-*g*-PSMA doped with three metal ions is porous. The Gel–15-Na^+^ system had the smallest micropore size (50–150 μm), followed by Gel–15-Mg^2+^ (200–400 μm), and Gel–15-Ca^2+^ max (250–500 μm). The change in the microstructure of the hydrogel may be due to the influence of metal ions on the morphology of the ADS micelles. When the ADS–MCl_*n*_ ionic micelle system with strong solubilization ability was used, a more compact and dense gel network was obtained.

Hydrogels with large pore sizes (>100 μm) are shown to be beneficial for cell ingrowth in bone regeneration applications. When the pore size of a hydrogel is similar to that of human bone (200–400 μm), it is suitable for cartilage formation.^49^ Thus, the pore size of the Gel–15-Mg^2+^ hydrogel is in the appropriate size range. In addition, the SEM images show that the surface of the hydrogel doped with Na^+^ and Mg^2+^ was smooth, whereas that of the hydrogel doped with Ca^2+^ was rough. The rough appearance of Gel–Ca^2+^ may be due to the insoluble calcium dodecyl sulfate in the hydrogel system.

### Swelling behavior

**Fig. 6:**
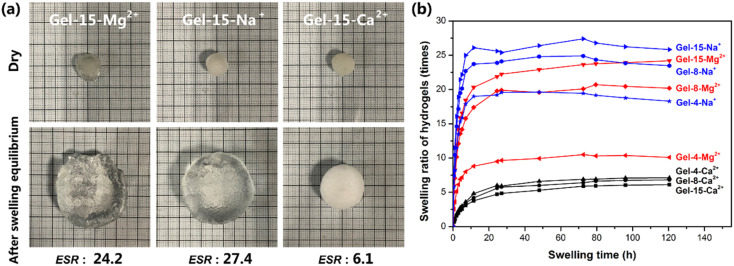
(a) Appearance of MCl_*n*_-doped PDMA-*g*-PSMA hydrogel before and after swelling. (b) Swelling curves of MCl_*n*_-doped PDMA-*g*-PSMA hydrogels.

The freeze-dried PDMA-*g*-PSMA hydrogel was soaked in distilled water to observe its swelling (Fig. 6a). Among the samples, the equilibrium swelling ratio of the PDMA-*g*-PSMA hydrogel doped with NaCl was the highest, and the ESR values of Gel–15-Na^+^, Gel–8-Na^+^ and Gel–4-Na^+^ were 27.4, 24.9 and 19.6, respectively. The MgCl_2_-doped PDMA-*g*-PSMA hydrogels had the next highest values, and the ESR values of Gel–15-Mg^2+^, Gel–8-Mg^2+^, and Gel–4-Mg^2+^ were 24.2, 20.7, and 10.5, respectively. The equilibrium swelling ratio of the CaCl_2_-doped PDMA-*g*-PSMA hydrogel was the lowest, and the ESR values of Gel–15-Ca^2+^, Gel–8-Ca^2+^ and Gel–4-Ca^2+^ were 6.1, 6.8 and 7.1, respectively (Fig. 6b). The swelling of hydrogels is a complex physical process. Factors such as salting-out effect, osmotic pressure, shielding electrostatic repulsion, hydrophobic interchain interaction, metal ion strength, and cross-linked network structure can affect the hydration of the PDMA-*g*-PSMA hydrophilic units with free water, thereby affecting the swelling kinetics.^50^ Na^+^ has a low charge and large hydration radius. The strong solubilization ability of ADS–NaCl ionic micelles can weaken the hydrophobic interaction between PSMA segments and improve the hydration efficiency of gels. As shown in Fig. 6b, the Gel–15-Na^+^, Gel–8-Na^+^ and Gel–4-Na^+^ hydrogels had the highest water absorption in the early stage, and could reach swelling equilibrium faster. However, the quality of the Gel–Na^+^ hydrogel began to decline after soaking for 72 h. This is because the Gel–Na^+^ hydrogel had strong medium permeability. The hydrophilic components such as NaCl and ADS will diffuse and be lost during the swelling process of the hydrogel. The loss of ADS–NaCl may cause the hydrogel to lose its self-healing function.^33^ Compared with Na^+^, Mg^2+^ has a higher chemical valence and smaller hydration radius. Simultaneously, the solubility of the ADS–MgCl_2_ ionic micelles for the PSMA segments was also lower than that of the ADS–NaCl system. Therefore, the swelling rate and equilibrium swelling rate of the Gel–Mg^2+^ hydrogel were lower than that of Gel–Na^+^. Due to the large molecular size of magnesium dodecyl sulfate and the high stability of ADS–MgCl_2_ micelles, no decrease in the quality of the Gel–Mg^2+^ hydrogel was observed at the later stage of swelling. In the case of the CaCl_2_-doped hydrogel system, the hydrophilicity of the gel was greatly reduced due to the combination of CaCl_2_ and sulfate ions. Simultaneously, the solubility of the ADS–CaCl_2_ system for the PSMA segments was very low, making Gel–Ca^2+^ more similar to a stable covalently crosslinked chemical gel in microstructure. Therefore, the equilibrium swelling rate of Gel–Ca^2+^ was the lowest (6.1–7.1).

**Fig. 7:**
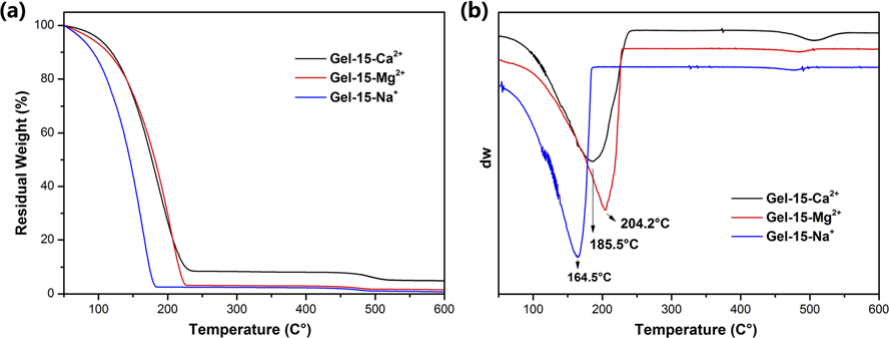
(a) TGA and (b) DTG curves of Gel–15-Na^+^, Gel–15-Mg^2+^ and Gel–15-Ca^2+^ hydrogels after swelling equilibrium.

The hydration of PDMA-*g*-PSMA hydrogel was analyzed by thermogravimetric analysis (Fig. 7). Generally, the water in hydrogels has three forms, as follows: (a) polarization with charged ion groups (such as NH^+^, COO^−^, and SO_4_^2−^) or fixation by hydrogen bond groups or other dipoles (bound water). (b) Distributed in the voids of the gel network (free water). (c) Surrounded by an “ice cage” formed by hydrophobic groups.^51^ It has been reported that as a hydrogel swells, the free water content increases, the bound water content decreases, and the intermediate water content remains essentially constant.^52^

According to the TGA and DTG curves (Fig. 7), the thermal weight loss process of the PDMA-*g*-PSMA hydrogel can be divided into two stages. In the first stage (50–240 °C), the weight loss of Gel–15-Na^+^, Gel–15-Mg^2+^ and Gel–15-Ca^2+^ was 97.48%, 96.82% and 91.50%, respectively. The weight loss at this stage is mainly contributed by the free water and bound water in the hydrogel. Mass spectrometry showed that when the temperature was below 150 °C, only water was lost. The fastest dehydration temperature of the three systems was determined by the peak in the DTG curve (position indicated by the arrow in Fig. 7b, Gel–15-Na^+^: 164.5 °C, Gel–15-Mg^2+^: 204.2 °C, and Gel–15-Ca^2+^: 185.5 °C). Therefore, the combination of Gel–15-Mg^2+^ and water is the most stable, and the dehydration temperature was the highest. The second weight loss stage was 300–550 °C. In this stage, the thermal decomposition of PDMA-*g*-PSMA polymer segments is the main process. The DTG curve showed that the decomposition temperature of PDMA-*g*-PSMA doped with Ca^2+^ is the highest, followed by Mg^2+^ doping, and Na^+^ doping is the lowest.

### Mechanical properties and self-healing properties

**Fig. 8:**
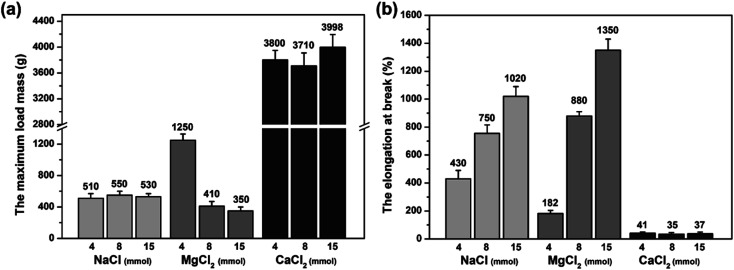
(a) Maximum load mass (*M*_max_) and (b) elongation at break of MCl_*n*_-doped PDMA-*g*-PSMA hydrogel.

The mechanical property of the MCl_*n*_-doped PDMA-*g*-PSMA hydrogel was characterized by loading weight. As can be seen from the data of the maximum load weight, *M*_max_ (Fig. 8a), and the elongation at break (Fig. 8b) of the hydrogel, the PDMA-*g*-PSMA hydrogels (Gel–4-Na^+^, Gel–8-Na^+^, Gel–15-Na^+^, Gel–8-Mg^2+^ and Gel–15-Mg^2+^) obtained by polymerization in the state of complete dissolution of SMA have slightly lower strength, but better elasticity. The PDMA-*g*-PSMA hydrogels (Gel–4-Mg^2+^, Gel–4-Ca^2+^, Gel–8-Ca^2+^ and Gel–15-Ca^2+^) obtained by polymerization in the state of incomplete dissolution of SMA have high strength but poor elasticity. In addition, the strength of the PDMA-*g*-PSMA hydrogels decreased and their elasticity increased with the addition of MCl_*n*_. For example, the *M*_max_ of Gel–Mg^2+^ hydrogel decreased from 1250 g to 350 g and the elongation at break increased from 182% to 1350% when the content of MgCl_2_ increased from 4 mmol to 15 mmol. Gel–8-Mg^2+^ is the complete dissolution system of SMA and Gel–4-Mg^2+^ is the incomplete dissolution system of SMA. This indicates that the mechanical properties of the hydrogels will change significantly when the solubility of the hydrophobic segments in the system changes. When ADS–MCl_*n*_ ionic micelles with high solubilization ability of hydrophobic monomer were selected, the dispersion of SMA in the water phase was more uniform, and the size of the hydrophobic crosslinking region of the PDMA-*g*-PSMA hydrogel obtained by polymerization was smaller. Simultaneously, the ability of the ADS–MCl_*n*_ micelles to dissolve PSMA segments can also weaken the strong hydrophobic interaction in the hydrogels, resulting in a decrease in their *M*_max_. When subjected to an external force, the weakening effect can generate an energy dissipation mechanism along the stress direction of the hydrogel, and thus the toughness of the product is remarkably improved.

Furthermore, four hydrogels with good elasticity, including Gel–8-Na^+^, Gel–15-Na^+^, Gel–8-Mg^2+^ and Gel–15-Mg^2+^, were selected for the tensile-recovery test. Namely, the hydrogel with a length of 3 cm was slowly stretched to 15 cm and kept for 2 min. Subsequently, the stretch was released and it was left to recover for 2 min at room temperature. The average length of the Gel–8-Na^+^, Gel–15-Na^+^, Gel–8-Mg^2+^ and Gel–15-Mg^2+^ hydrogels was 3.9 cm, 4.5 cm, 3.4 cm and 3.7 cm, respectively, after 10 cycles of stretching and recovery. It can be seen that properly reducing the doping concentration of MCl_*n*_ can prolong the life of hydrophobic association and reduce the irreversible deformation of hydrogel under external force. Meanwhile, compared with the monovalent Na^+^ doping, the divalent Mg^2+^ doping resulted in the formation of ADS–MCl_*n*_ micellar aggregates with higher rigidity and better stability, thereby improving the shape stability of the PDMA-*g*-PSMA hydrogel.

**Fig. 9:**
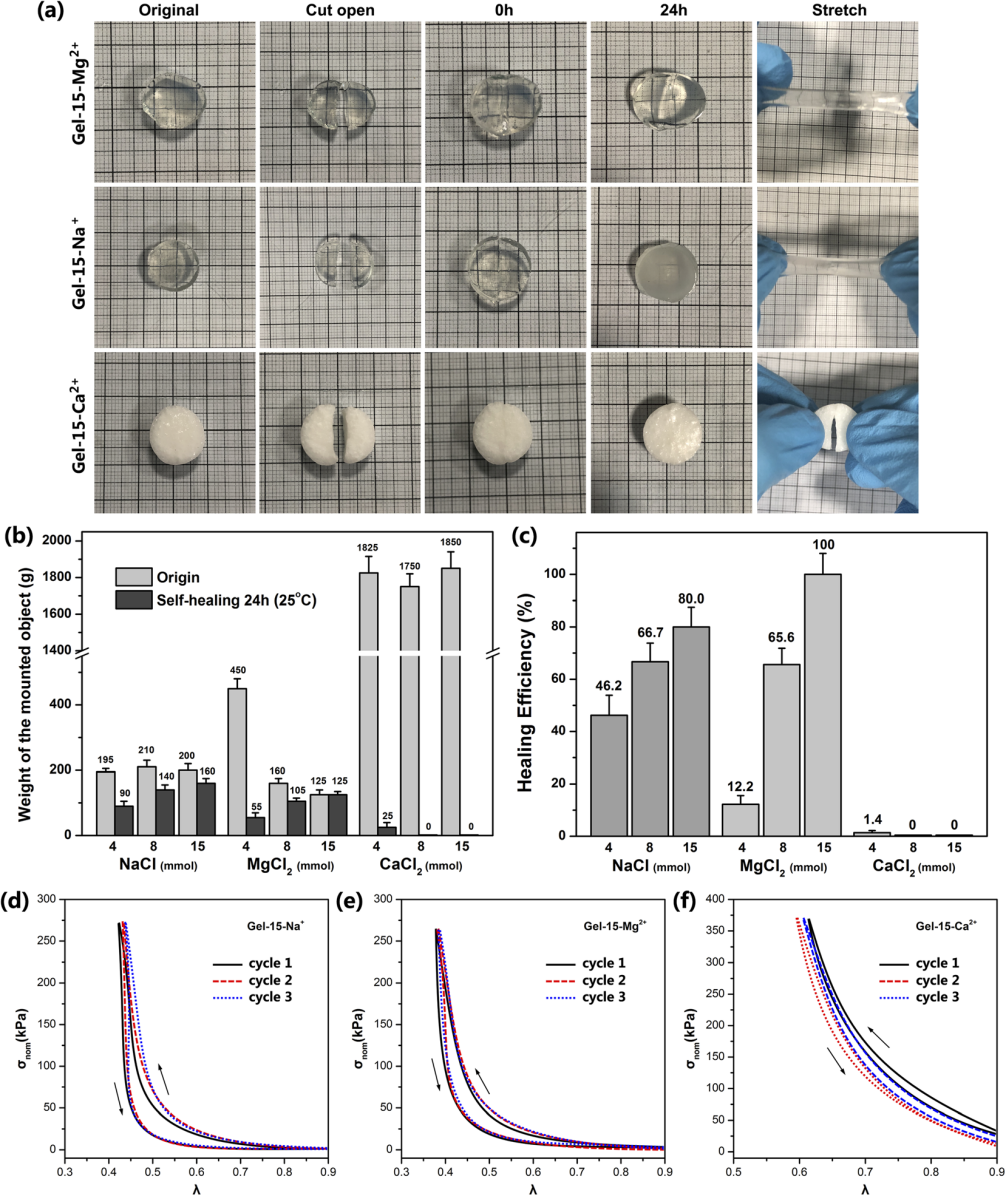
(a) Photos of self-healing process of Gel–15-Na^+^, Gel–15-Mg^2+^ and Gel–15-Ca^2+^ hydrogels. (b) Maximum loading mass of MCl_*n*_-doped PDMA-*g*-PSMA hydrogel before and after self-healing. (c) Self-healing efficiency of MCl_*n*_-doped PDMA-*g*-PSMA hydrogel at 24 h. Stress–strain curves of (d) Gel–15-Na^+^, (e) Gel–15-Mg^2+^, and (f) Gel–15-Ca^2+^ hydrogels under three successive loading/unloading cycles.

The self-healing behavior of the MCl_*n*_-doped PDMA-*g*-PSMA hydrogel was studied by the visual observation and loading weights. The hydrogel was cut in half, and then its cross-section was re-bonded. After 24 h at room temperature, it was found that the incisions of Gel–4-Na^+^, Gel–8-Na^+^, Gel–15-Na^+^, Gel–8-Mg^2+^ and Gel–15-Mg^2+^ were almost completely healed and had good tensile strength. The cross sections of Gel–4-Mg^2+^, Gel–4-Ca^2+^, Gel–8-Ca^2+^ and Gel–15-Ca^2+^ only exhibited a small amount of self-healing and no ductility (Fig. 9a). The self-healing efficiency (*M*_sh_/*M*_o_) of the MCl_*n*_-doped PDMA-*g*-PSMA hydrogels was quantitatively characterized by loading weights. The self-healing efficiency of the Gel–15-Mg^2+^ hydrogel was the highest. The maximum loading mass before and after repair was 125 g, and the self-healing efficiency reached 100%. Gel–15-Na^+^ exhibited the second highest self-healing efficiency, reaching 80%. The Gel–Ca^2+^ series was the worst, which exhibited the self-healing efficiency of only 0–1.4%. In addition, with a reduction in the amount of NaCl and MgCl_2_, it was observed that the self-healing efficiency of the hydrogel was significantly reduced. This indicates that the self-healing process of the PDMA-*g*-PSMA hydrogels depends heavily on the solubilization of the hydrophobic segments by the ADS–MCl_*n*_ ionic micelles. When the ADS–MCl_*n*_ ionic micelles effectively solubilize the PSMA hydrophobic segments, the physical cross-linking of the hydrophobic PSMA segments randomly distributed along the main chain of the hydrophilic polymers in hydrogels has high reversibility. When the hydrogel is damaged by external force, the strong hydrophobic interaction and solubilization of the ADS–MCl_*n*_ ionic micelles can generate the hydrophobic association again between the damaged surfaces to achieve self-healing of the hydrogel at room temperature. When the ADS–MCl_*n*_ system could not solubilize the PSMA segments, the mechanical properties of PDMA-*g*-PSMA were similar to that of the covalently crosslinked chemical hydrogels. Due to the lack of effective energy dissipation mechanism in its gel network, its resistance to crack propagation was very low, and thus it could not exhibit the self-healing function.

The mechanical behavior of the MCl_*n*_-doped PDMA-*g*-PSMA hydrogel under stress was characterized by a uniaxial compression test. Fig. 9d–f show the stress–strain curve of the Gel–15-Na^+^, Gel–15-Mg^2+^ and Gel–15-Ca^2+^ hydrogels under three successive loading/unloading cycles, with an interval of 2 min for each cycle, respectively. In all cases, the loading curve and unloading curve of the compression cycle do not coincide, which indicates that damage and energy dissipation occurred in the compression process of the hydrogel. Among them, the three loading curves of Gel–15-Na^+^ and Gel–15-Mg^2+^ had high coincidence, which indicates that the damage to the Gel–15-Na^+^ and Gel–15-Mg^2+^ hydrogels during the loading cycle was essentially recoverable. Gel–15-Na^+^ and Gel–15-Mg^2+^ have a reversible dynamic crosslinking structure. In contrast, the three loading curves of the Gel–15-Ca^2+^ hydrogel have obvious deviation, which indicates that the Gel–15-Ca^2+^ hydrogel had irreversible damage during the loading cycle. This conclusion is consistent with the characterization results of self-healing efficiency.

### Contact angle and friction properties

**Fig. 10:**
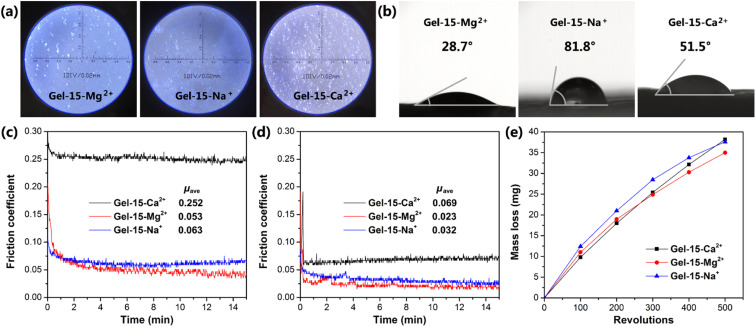
(a) Optical microscopy images and (b) photo of contact angle of Gel–15-Mg^2+^, Gel–15-Na^+^ and Gel–15-Ca^2+^ hydrogels. Friction coefficient curves of Gel–15-Na^+^, Gel–15-Mg^2+^, and Gel–15-Ca^2+^ hydrogels before (c) and after (d) water lubrication. (e) Mass loss curves of Gel–15-Na^+^, Gel–15-Mg^2+^ and Gel–15-Ca^2+^ hydrogel films under rubber-wheel abrasion.

The surface morphology of the MCl_*n*_-doped PDMA-*g*-PSMA hydrogel was observed using an optical microscope. As shown in Fig. 10a, the surfaces of the Gel–15-Na^+^ and Gel–15-Mg^2+^ were relatively flat with few defects. In contrast, the surface of the Gel–15-Ca^2+^ hydrogel was rough, which can be attributed to the precipitation of calcium dodecyl sulfate. The surface hydrophilicity of the MCl_*n*_-doped PDMA-*g*-PSMA hydrogel was characterized by contact angle measurements. The results show (Fig. 10b) that the water contact angles of the Gel–15-Na^+^, Gel–15-Mg^2+^, and Gel–15-Ca^2+^ hydrogels are 28.7°, 81.8°, and 51.5°, respectively. Gel–15-Mg^2+^ had the highest hydrophilicity, followed by Gel–15-Ca^2+^, and Gel–15-Na^+^ had the lowest. The surface hydrophilicity of the PDMA-*g*-PSMA hydrogel is related to the distribution of hydrophilic segments in the copolymer and the surface roughness of the hydrogel. The hydrophilicity and hydrophobicity of the hydrogel surface may have a certain impact on the adhesion of chondrocytes and the lubrication of artificial cartilage by body fluid.

The friction behavior of the hydrogel was characterized using a CFT-1 material surface performance tester. Fig. 10c shows the friction curve of the PDMA-*g*-PSMA hydrogel under 3 N load. The surface friction of the hydrogel was high in the period of 0–1 min. As the test time was prolonged, the friction coefficient gradually decreased and tended to be stable. This is because the friction behavior will promote the release of free water on the surface of the PDMA-*g*-PSMA hydrogel, resulting in a self-lubricating effect. In the initial stage of friction, the amount of water overflowing from the surface of the hydrogel was low, and the self-lubricating function was not significant. Therefore, the measured friction coefficient was larger. *μ*_ave_ is defined as the average friction coefficient in the period of 2–15 min. The *μ*_ave_ of the Gel–15-Na^+^, Gel–15-Mg^2+^ and Gel–15-Ca^2+^ hydrogels was 0.063, 0.053 and 0.252, respectively. The friction coefficient of the MgCl_2_-doped PDMA-*g*-PSMA hydrogel was the lowest. This can be attributed to the fact that its high hydrophilicity promotes the wetting of free water on the hydrogel surface and strengthens the self-lubricating effect. The Gel–15-Ca^2+^ hydrogel had a high friction coefficient due to its high dehydration issue (according to TGA test results) and large surface roughness.

The surface lubrication of the hydrogel with exogenous water can simulate the lubrication function of body fluid on articular cartilage. Fig. 10d shows the friction curve of the hydrogel after water lubrication. The data shows that the *μ*_ave_ of the Gel–15-Na^+^, Gel–15-Mg^2+^ and Gel–15-Ca^2+^ hydrogels was significantly reduced to 0.032, 0.023 and 0.069, respectively, by using water as a lubricant. This indicates that water molecules will be brought into the wear surface during the friction process, and better lubrication will be achieved between the friction pairs. There are many factors affecting the lubrication process of hydrogels, including the physical and chemical characteristics of the surface, the deformability of the surface, and the stress-induced water transport behavior of the gel network. According to the repulsion-adsorption model of hydrogel friction, the friction of a hydrogel can be affected by the electrostatic interaction between the surface charges or the adsorption–desorption process of the polymer chains at the interface. When there is a repulsive interaction between the polymer chains on the hydrogel surface and the sliding object, a liquid film will be formed between the shear bodies, thus promoting lubrication and reducing friction. In addition, the ionization behavior and migration behavior of the PDMA-*g*-PSMA hydrogel in water also have an impact on its friction behavior.

Considering that there is no significant correlation between the friction coefficient of hydrogel and wear,^25^ it is also necessary to study the wear properties of PDMA-*g*-PSMA. The mass loss of the MCl_*n*_-doped PDMA-*g*-PSMA hydrogel during wear was tested using the rotating abrasive rubber wheel method (Fig. 10e). To reduce the interference of free water volatilization on the test results, the wear test was carried out at 20 °C and 85% humidity. As shown in Fig. 10e, the mass loss of the Gel–15-Ca^2+^ hydrogel was lower than that of Gel–15-Na^+^ and Gel–15-Mg^2+^ in the initial stage of the wear test. This is due to the better mechanical strength of the Gel–15-Ca^2+^ hydrogel. The mass loss of Gel–15-Na^+^ and Gel–15-Mg^2+^ began to decrease when the wear test was carried out over 200 revolutions. It is speculated that the Gel–15-Na^+^ and Gel–15-Mg^2+^ hydrogels completed the release of free water at this stage, reducing the friction loss of the polymers through self-lubrication. In contrast, the wear mass loss of the Gel–15-Ca^2+^ hydrogel increased linearly with an increase in the number of wear cycles and the antifriction process caused by self-lubrication was not observed. After 500 cycles of wear test, the mass loss of the Gel–15-Na^+^, Gel–15-Mg^2+^ and Gel–15-Ca^2+^ hydrogels was 37.6 mg, 35.0 mg and 38.2 mg, respectively.

In summary, the Gel–15-Mg^2+^ hydrogel showed the relatively best friction reduction and wear resistance under load in a number of test systems. With water as the lubricant, the friction coefficient of the Gel–15-Mg^2+^ hydrogel reached 0.023, which is lower than the upper limit of the friction coefficient of natural cartilage (0.03).

### Cytotoxicity test

**Fig. 11:**
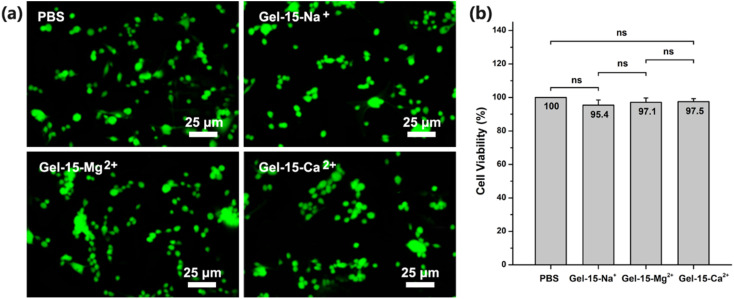
(a) Fluorescence image (stained with calcein/propidium iodide) and (b) cell viability of L929 cells cultured in PBS and different hydrogel extracts for 1 day *in vitro*.

The cytotoxicity of the MCl_*n*_-doped PDMA-*g*-PSMA hydrogel was studied using the standard CCK-8 assay. After 24 h of co-culture, the mouse L929 fibroblasts were stained with calcein (AM, green, for live cells) and propidium iodide (PI, red, for dead cells). As shown in Fig. 11a, the images of the PDMA-*g*-PSMA hydrogel culture group were green under doping with different metal ions, indicating that there is a large number of living cells in the system. The survival rate of L929 cells cultured with Gel–15-Na^+^, Gel–15-Mg^2+^ and Gel–15-Ca^2+^ was 95.4%, 97.1% and 97.5%, respectively (Fig. 11b). The results of significance analysis showed that there was no significant difference in the survival rate among the culture groups. Therefore, the PDMA-*g*-PSMA hydrogels doped with NaCl, MgCl_2_ and CaCl_2_ have good biocompatibility.

## Conclusions

Herein, hydrophobic association MCl_*n*_-doped PDMA-*g*-PSMA hydrogels were prepared *via* the UV crosslinking method, using NaCl/MgCl_2_/CaCl_2_ as doped inorganic salts, ADS as ionic micelles and Irgacure-2959 as the photoinitiator. This method has the advantages of high polymerization efficiency, less monomer residue, convenient operation and easy molding, which is an efficient way for preparing artificial articular cartilage *in situ*. Subsequently, the micro morphology, mechanical properties, self-healing properties, friction properties and cytotoxicity of the hydrogels were studied. The main conclusions are as follows.

(1) After doping with CaCl_2_, Ca^2+^ will cause the crystallization and precipitation of the anionic surfactant. SMA will participate in the polymerization in the form of monomer droplets. The obtained PDMA-*g*-PSMA hydrogels have large pores, rough surface, high tensile strength, low elongation at break and no self-healing function.

(2) With the addition of NaCl or MgCl_2_, Na^+^ or Mg^2+^ will combine with the micelles, making the diffusion double electric layer structure of the ionic micelle thinner. This process will promote the micelles to grow from spherical to rod-shaped, large cylindrical aggregates or flexible worm-shaped micelles, which improves the solubilization ability of the ADS–MCl_*n*_ micelles for the SMA monomer and PSMA chain segments. The obtained PDMA-*g*-PSMA hydrogel has reversible hydrophobic association with limited service life, is uniform and compact, has high elongation at break, good elasticity, large equilibrium swelling ratio and good self-healing function.

(3) The solubilization effect of ionic micelles on PSMA can be improved by increasing the doping amount of Na^+^ or Mg^2+^, which makes the *M*_max_ of PDMA-*g*-PSMA hydrogel decrease, and the elongation at break, ESR and self-healing efficiency increase.

(4) Compared with monovalent Na^+^ doping, the PDMA-*g*-PSMA hydrogel obtained by divalent Mg^2+^ doping had higher self-healing efficiency (100%), better the shape stability, stronger hydrophilicity, lower friction coefficient (0.023), and lower wear loss (35.0 mg). In addition, no component loss was observed in the swelling process of Gel–Mg^2+^.

(5) The PDMA-*g*-PSMA hydrogels obtained from the three doping systems have good biocompatibility, which can be utilized as antifriction materials for the study of artificial articular cartilage.

## Conflicts of interest

There are no conflicts to declare.

## Supplementary Material
